# A Prospective Study on the Relationship between Iron Supplement Intake, Hemoglobin Concentration, and Risk of Parkinsonism

**DOI:** 10.3390/nu14214671

**Published:** 2022-11-04

**Authors:** Hikaru Takeuchi, Ryuta Kawashima

**Affiliations:** 1 Division of Developmental Cognitive Neuroscience, Institute of Development, Aging and Cancer, Tohoku University, 4-1 Seiryo-cho, Aoba-ku, Sendai 980-8575, Japan; 2 Smart Aging Research Center, Tohoku University, Sendai 980-8575, Japan; 3 Department of Advanced Brain Science, Institute of Development, Aging and Cancer, Tohoku University, Sendai 980-8575, Japan

**Keywords:** iron supplement, hemoglobin, parkinsonism, prospective study

## Abstract

The findings regarding whether the greater iron level or intake is a risk factor to Parkinson’s disease (PD) or parkinsonism was not clear. The purpose of this study is to establish a consistent association between iron supplementation and parkinsonism risk, we conducted a large-scale prospective cohort study using comprehensive longitudinal data from the UK Biobank. The longitudinal cohort data of 385,898 participants (including 911 cases) who were middle to old aged British adults and joined the UK Biobank study from 2006 to 2010 and were followed up until 2018 was analyzed. The associations between iron supplement intake, hemoglobin levels and all cause subsequent parkinsonism risk after corrections of potential confounders (sex, age, household income, education length, employment status, deprivation level, body mass index, physical activity level, household numbers, smoking and drinking levels, health status, blood pressure) were investigated. Analyses revealed that (a) iron supplementation was significantly associated with higher parkinsonism risk, (b) greater hemoglobin was weakly and insignificantly associated with lower parkinsonism risk, and (c) multivitamin or vitamin C supplement intake was not significantly associated with parkinsonism risk. Regardless of whether the subjects were classified as anemic, normal, or polycythemic or in the hemoglobin level quintile, there was no nonlinear association between hemoglobin and parkinsonism risk. Parkinsonism risk did not differ between participants reporting supplementary iron intake with or without vitamin C or multivitamin supplement intake. Furthermore, polygenic risk score of PD negatively correlated with hemoglobin level, while it did not associate with intake of iron supplement or multivitamin or vitamin C supplement intake. The results suggest excessive iron intake may increase parkinsonism risk. Interventional studies are warranted to examine whether iron intake restriction is beneficial for individuals without clinical iron deficiency.

## 1. Introduction

Parkinson’s disease (PD) is a progressive neurological disorder caused by the degeneration of dopaminergic neurons in the substantia nigra (SN) [1]. It is more prevalent in middle-aged and older individuals (3.5% of Europeans aged 85–89 years [2]) and is characterized primarily by impaired motor functions but may progress to include other neurological deficits, including cognitive dysfunction [1]. Pathological diagnosis of PD requires the detection of inclusion bodies termed Lewy bodies composed of the synaptic protein alpha-synuclein in the SN [3]. Thus, the umbrella term parkinsonism is used to describe adult-onset motor symptoms of PD regardless of pathological confirmation [3], and the prevalence in this study cohort is 5.1%.

Iron dysregulation is believe to be a major pathomechanism for PD and parkinsonism [4]. While iron is critically important for dopamine synthesis [4] in the SN, deposits of excess iron are found in the SN of patients with PD and in animal models of dopaminergic neuronal degeneration [5]. In addition, the SN of parkinsonism patients contains pathological Lewy bodies [6], and iron has been shown to influence the synthesis, post-translation modification, and aggregation of α-synuclein [7]. Moreover, iron chelation has been shown to protect against degeneration and motor dysfunction in PD model mice [8], whereas a cross-sectional study of PD patients found that iron supplements led to faster disease progression [9].

However, a Mendelian randomization study suggested that serum iron may protect against PD [10], and anemia due to low iron is associated with increased lifetime risk of PD [11,12]. Iron deficiency is also associated with motor dysfunction [13]. Still other studies have reported that high levels of iron in the diet reduce PD risk [14,15], while other studies showed the increased risk [16]. A variety of factors may explain these discrepancies. For instance, Logroscino and colleagues reported that total iron intake was not associated with increased risk of PD, but that dietary nonheme iron intake from food was associated with increased risk of PD in subjects with low vitamin C intake [17]. In relation to this, vitamin C facilitates iron absorption in the small intestine and enhances absorption of soluble nonheme iron [18].

These inconsistences have left many outstanding questions to be resolved. And to resolve these we set the following hypotheses based on the aforementioned previous studies. Hypothesis 1: Iron supplement intake is associated with increased risk of parkinsonism. This main hypothesis is based on animal studies associating iron excess with PD risk [8] and some divided human studies of the effects of greater iron intake on PD risk [16] or progression risk in PD patients [9]. Hypothesis 2: Both increased and decreased hemoglobin levels are associated with risk of parkinsonism. This hypothesis is based on the dopamine system that sometimes demonstrates an inverted U-shaped relationship [19]. There are also conflicting previous studies that associated both iron deficiency and extremely high iron intake with PD risk. Hypothesis 3: The risk of parkinsonism is reduced when vitamin C is taken together with supplementary iron. This hypothesis is based on the aforementioned study of iron intake from diet [17]. Hypothesis 4: Low hemoglobin level without intake of supplemental iron does not necessarily increase the risk of parkinsonism. Hypothesis 5: Supplementary iron intake is associated with increased risk of parkinsonism in the absence of pathological conditions necessitating this intake. Hypotheses 4 and 5 are based on the aforementioned studies of the association between anemia and PD risk [11,12] as well as some studies linking higher iron intake with PD risk [16].

This study aimed to test these hypotheses. And for this purpose, we utilized large-scale longitudinal data on incident parkinsonism from the UK Biobank, which includes the medical records of over 500,000 participants. The scope of the data included allows for the analyses of a wide range of relationships while controlling for many covariates.

## 2. Methods

### 2.1. Participants

We used data from the UK Biobank, a large-scale database established from a prospective cohort study of middle-aged individuals in the United Kingdom. The procedures for subject recruitment and data gathering are described elsewhere (http://www.ukBiobank.ac.uk/wp-content/uploads/2011/11/UK-Biobank-Protocol.pdf, accessed on 5 July 2021). Briefly, adults aged 40–69 years old who were registered with the National Health Service and lived within 25 miles from the study evaluation sites were invited via email to take part in the UK Biobank. No exclusion criteria were applied for recruitment. The present study was approved by the North-West Multi-center Research Ethics Committee (Manchester, UK), and written informed consent was obtained from each participant. Briefly, participants went to one of 22 assessment centers throughout the UK for data collection, and baseline data were obtained from 502 505 participants. The present study included baseline data obtained at the first assessment visit (2006–2010). The following analyses included only complete individual subject data (i.e., all relevant dependent and independent variables, including genetic variables). The descriptions in this subsection are largely reproduced from our previous study using the same methods [20].

### 2.2. Supplemental Iron Intake

Iron supplement intake was evaluated by the following question. “Do you regularly take any of the following? (You can select more than one answer)” (Data field 6179). Response options were “Iron”, “Selenium”, “Zinc”, “Calcium”, “Glucosamine”, “Fish oil (including cod liver oil)”, “None of the above,” and “Prefer not to answer.” Subjects without data for this item and those who chose “Prefer not to answer” were excluded from the relevant analyses.

### 2.3. Intake of Vitamin C or Multivitamins

Supplementary intake of other vitamins was evaluated by the following question. “Do you regularly take any of the following? (You can select more than one answer)” (Data field 6155). The possible responses included “Vitamin A,” “Vitamin B,” “Vitamin C,” “Vitamin D,” “Vitamin E,” “Folic acid or Folate (Vit B9),” “Multivitamins +/- minerals,” “None of the above”, and “Prefer not to answer.” Subjects without data for this item and those who chose “Prefer not to answer” were excluded from the relevant analyses.

### 2.4. Serum Hemoglobin Measurements

Serum hemoglobin concentration (Field ID 30020) was measured at the central processing laboratory of the UK Biocenter. A median of one measurement was obtained per subject. Hemoglobin concentration was measured in EDTA-anticoagulated blood using the Sysmex XN-1000 hematology analyzer. The descriptions in this subsection are reproduced from a previous study using the same data [21].

Associations with other variables such as incident parkinsonism were analyzed in three ways. First, serum hemoglobin concentration was included as a continuous variable. Second, concentrations were stratified into 5 quintiles. Third, we divided concentrations into anemia, normal range, and polycythemia according to criteria of World Health Organization [22,23]: anemia (women: <115 g/L, men: <130 g/L), polycythemia (women: >160 g/L, men: >165 g/L). The latter two methods were used to investigate the nonlinearity of the association between hemoglobin concentration and risk of incident parkinsonism.

### 2.5. Sociodemographic and Lifestyle Measurements Used as Covariates

From the database, neighborhood-level socioeconomic status at recruitment (cov1), education level at recruitment (cov2), household income (cov3), current employment status (cov4), body mass index (cov5), metabolic equivalent of task hours (cov6), number of people in the household (cov7), current tobacco smoking level (cov8), current alcohol drinking unit (cov9), systolic blood pressure (cov10), ethnic group (white or not) (cov11), and overall health rating (cov12) were included as common covariates across analyses along with sex (self-reported) and age at baseline as described below. For additional details, refer to Supplemental Methods. The descriptions in this subsection are largely reproduced from our previous study using the same methods [20].

### 2.6. Statistical Analyses

All statistical analyses were performed using Predictive Analysis Software, version 22.0.0 (SPSS Inc., Chicago, IL, USA; 2010). The descriptions in this subsection are reproduced from our previous study using similar methods [20].

Cox proportional hazard models were constructed to examine the relationships of iron supplement intake and other factors at the first assessment visit with subsequent incident all-cause parkinsonism while controlling for various covariates. All-cause parkinsonism was ascertained using hospital inpatient records and linkage to death registry data. This method to determine the incident of certain diseases from the hospital inpatient record and linkage to death registry data has been employed in representative studies of this field using the UK Biobank data [24]. For more details on identification of incident parkinsonism, see Supplemental Methods. Subjects in the UK Biobank database already diagnosed with parkinsonism at baseline assessment, with self-reported PD at baseline, or with self-reported parkinsonism without a diagnosis in either hospital inpatient records or death registry data were excluded from the current analyses. The time period considered for incident parkinsonism spanned from the first assessment visit (2006–2010) until February 28, 2018.

Analysis 1: In the first analysis, self-reported sex, age at first assessment visit, cov1–cov12 values at first assessment visit, iron supplement intake status at the first assessment visit, intake of multivitamin or vitamin C at the first assessment visit, and hemoglobin concentration were included in the Cox proportional hazard model with all-cause parkinsonism as the dependent variable.

Analysis 2: We then repeated this analysis but with hemoglobin concentration removed as it can be influenced by iron supplement intake.

Analysis 3: We constructed another model, including age at first assessment, sex, cov1–12, and the 4-category variable combining iron supplement intake (Yes/No) and intake of multivitamin or vitamin C (Yes/No).

Analysis 4: The fourth model included age at first assessment, sex, cov1–12, and hemoglobin concentration level but not iron supplement intake and vitamin C or multivitamin intake.

Analysis 5: We conducted an analysis in which the model included age at first assessment, sex, cov1–11 (but not cov12), and hemoglobin concentration but not iron supplement intake and vitamin C or multivitamin intake. Health rating (cov12) was excluded to evaluate whether the previously reported robust association between hemoglobin and incident parkinsonism is mediate by general health.

Analysis 6: The sixth model included age at first assessment, sex, cov1–12, and hemoglobin concentration level but not vitamin C or multivitamin intake among subject without iron supplement intake to investigate the effects of hemoglobin on parkinsonism independent of modulation by iron supplements.

Analysis 7 and 8. To evaluate the nonlinearity of hemoglobin level effects on all-cause parkinsonism, we constructed models, including age at first assessment, sex, cov1–12, and either the three-category hemoglobin variable of anemia, normal, and polycythemia (analysis 7) or the 5-category variable of quintiles (analysis 8) but not iron supplement intake and vitamin C or multivitamin intake.

### 2.7. Sensitivity Analyses Evaluating the Impact of Comorbidities on the Association between Iron Supplement Intake and Incident Parkinsonism

Next, we investigated if the association between iron supplement intake and incident parkinsonism risk is influenced by cancer and other serious medical conditions/disabilities (Analysis 9) or by self-reported anemia (Analysis 10). For Analysis 9, we removed subjects with a medical diagnosis of cancer (UK Biobank data field ID:2453) or other serious medical conditions/disabilities (UK Biobank data field ID:2473) and included the same covariates as in the main analyses. In addition, we repeated the analysis with the same covariates as well as iron supplement intake and vitamin C or multivitamin intake. For analysis 10, we removed subjects with self-reported anemia. For this selection, we used data of noncancer illness (UK Biobank data field ID:20002) and removed subjects who selected one of “aplastic anemia” (code: 1332), “pernicious anemia” (code: 1331), “iron deficiency anemia” (code:1330), or “anemia” (code:1440). We then repeated the analysis with the same covariates as in the main analysis but now including iron supplement intake and vitamin C or multivitamin intake. 

### 2.8. Corrections for Multiple Comparisons

Results with a threshold *p* < 0.05 corrected for false-discovery rate (FDR) using the two-stage sharpened method [25] were considered statistically significant. This correction was applied to the results of the 15 predictors for the 10 analyses described. In models with 3 to 5 categorical variables, only the *p* values for group differences and not post hoc *p* values were FDR corrected.

### 2.9. Polygenic Risk Score Analysis

Finally, we conducted polygenic risk score (PRS) analysis to examine if the genetic predisposition toward PD is associated with intake of iron supplement, intake of vitamin C or multivitamins, and hemoglobin concentration. We used PRS of PD instead of all-cause parkinsonism for this analysis because established results of genome-wide association studies are available. We utilized the summary statistics of Nalls, et al. [26] based on 1805 single nucleotide polymorphisms (SNPs) that best differentiated PD from controls in this previous study. This study is a meta-analysis of 17 GWAS datasets of PD using 37,688 patients, 18,618 proxy cases (i.e., First-degree relative of a PD patient but not a PD patient), and 1.4 million controls. From various thresholds, this study identified a threshold at which the PRS can be calculated that can better distinguish between patients and controls, which involved 1805 genetic polymorphisms. These SNPs were weighted based on the strengths of associations and the weights summed. For this calculation, we used the data of genetic Caucasoids (UK Biobank Data field 22006) after excluding data not meeting quality control and data from first-degree relatives of PD patients included in Nalls, Blauwendraat, Vallerga, Heilbron, Bandres-Ciga, Chang, Tan, Kia, Noyce and Xue [26]. For other details, see Supplemental Methods.

To investigate the associations between PRS of PD and variables of interest (namely, intake of iron supplement, intake of vitamin C or multivitamins, and hemoglobin concentration), we used partial correlation analyses (hemoglobin concentration) and multiple logistic regression analyses (supplement) controlling for age, sex, 10 genetic principal components (UK Biobank data field: 22009) and cov1–10 and 12 (excluding ethnicity).

## 3. Results

### 3.1. Baseline Characteristics of the Study Population

Baseline clinicodemographic characteristics of participants with and without incident all-cause parkinsonism are summarized in Table 1 and Table 2. Simple correlation coefficients between the main study variables (iron supplement intake, intake of vitamin C or multivitamins, and hemoglobin concentration) and other clinicodemographic values included as covariates in multiple regression analyses were all < 0.2 at baseline except that between sex and hemoglobin concentration (r = 0.607).

### 3.2. Prospective Analysis of All-Cause Parkinsonism

Among 502 505 individual datasets from the UK Biobank, data from 385,898 subjects were included in prospective analyses of incident all-cause parkinsonism. Parkinson’s disease was self-reported by only 132 participants, whereas 807 were already diagnosed with all-cause parkinsonism at first assessment. The remaining participants included 1329 cases of incident all-cause parkinsonism. Main analyses were performed on the 911 cases of all-cause parkinsonism and the 385 898 participants without incident parkinsonism for which all covariates were available. All analyses except for sensitivity analysis excluding certain subjects with comorbidities were performed using this data set of 385,898 participants.

**Figure 1:**
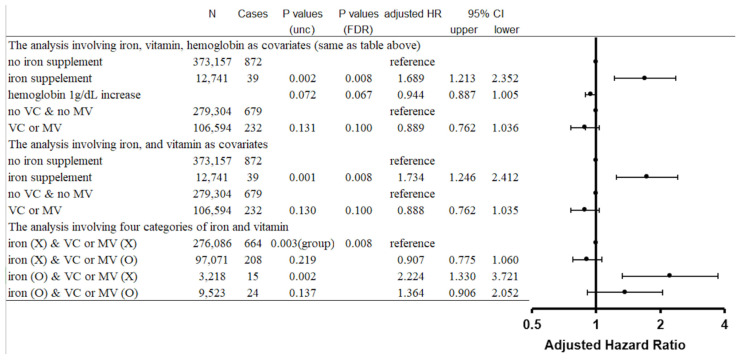
Statistical values (including hazard ratios [HRs] with 95% confidence intervals [95% CIs]) for the associations of all-cause parkinsonism with iron supplement intake, vitamin C or multivitamin intake, and serum hemoglobin concentration.

The Cox proportional hazard model, including iron supplement intake, vitamin C or multivitamin intake, and hemoglobin concentration as covariates, revealed that (a) iron supplement intake was significantly associated with the risk of incident parkinsonism (*p* = 0.002, FDR corrected *p* = 0.008, adjusted HR = 1.689, confidence interval [CI]: 1.213–2.352), (b) greater hemoglobin concentration was weakly and marginally insignificantly associated with lower risk of incident parkinsonism (*p* = 0.072, FDR corrected *p* = 0.067, adjusted HR [by 1 g/dL increase of hemoglobin concentration] = 0.944, CI: 0.887–1.005), and (c) intake of vitamin C or multivitamins was not significantly associated with altered risk of incident parkinsonism (*p* = 0.131, FDR corrected *p* = 0.100, adjusted HR = 0.889, CI:0.762–1.036) (Figure 1). The Cox proportional hazard model, including iron intake and vitamin C or multivitamin intake but not hemoglobin concentration as covariates, revealed similar results, with significant effects of iron intake and insignificant effects of vitamin C or multivitamin intake on incident parkinsonism (Figure 1).

The Cox proportional hazard model, including the 4-category variable combining iron intake (Yes/No) and vitamin C or multivitamin intake (Yes/No), revealed significant group differences (*p* = 0.003, FDR corrected *p* = 0.008), and post hoc analysis showed increased risk of incident parkinsonism in the group reporting iron supplement intake without vitamin C or multivitamin intake compared to the group reporting no iron supplement intake and no intake of vitamin C or multivitamins [*p* (uncorrected post hoc comparisons) = 0.002, adjusted HR = 2.224, CI:1.330–3.721] and compared to the group reporting no iron intake but confirming vitamin C or multivitamin intake [*p* (uncorrected post hoc comparisons) = 0.001, adjusted HR = 2.454, CI:1.449–4.155)]. In contrast, the difference between the group reporting iron intake plus vitamin C or multivitamin intake and the group reporting iron intake but no vitamin C or multivitamin intake did not reach significance [*p* (uncorrected post hoc comparisons) = 0.138]. Also, the difference between the group reporting iron intake plus vitamin C or multivitamin intake and the two groups reporting no iron intake (with or without vitamin C or multivitamin intake) did not reach significance (*p* = 0.059 and *p* = 0.137, respectively).

### 3.3. Effect of Hemoglobin Concentration on the Association between Supplementary Iron Intake and Incident Parkinsonism

As described, the effect of hemoglobin concentration on incident parkinsonism risk did not reach significance in analyses, including iron supplement intake and vitamin C or multivitamin intake (*p* = 0.072). However, the effect of hemoglobin concentration on incident parkinsonism risk was significant when iron supplement intake and vitamin C or multivitamin intake were excluded as covariates (*p* = 0.045, FDR corrected *p* = 0.047, adjusted HR [by 1 g/dL increase of hemoglobin concentration] = 0.938, CI: 0.881–0.998). This may be due to a negative correlation between iron intake and hemoglobin concentration. Indeed, partial correlation analysis correcting for covariates 1–12 described in Methods revealed a significant negative correlation between hemoglobin concentration and iron supplement intake (partial correlation coefficient = −0.054, *p* < 0.001). However, the effect of hemoglobin concentration on incident parkinsonism risk was significant among participants reporting no supplemental iron intake (*p* = 0.042, FDR corrected *p* = 0.047, adjusted HR [by 1 g/dL increase of hemoglobin concentration] = 0.935, CI: 0.877–0.998). The effect of hemoglobin concentration on incident parkinsonism risk was also significant when iron supplement intake, vitamin C or multivitamin intake, and overall health rating were excluded (*p* = 0.006, FDR corrected *p* = 0.010, adjusted HR [by 1 g/dL increase of hemoglobin concentration] = 0.915, CI: 0.859–0.975).

**Figure 2:**
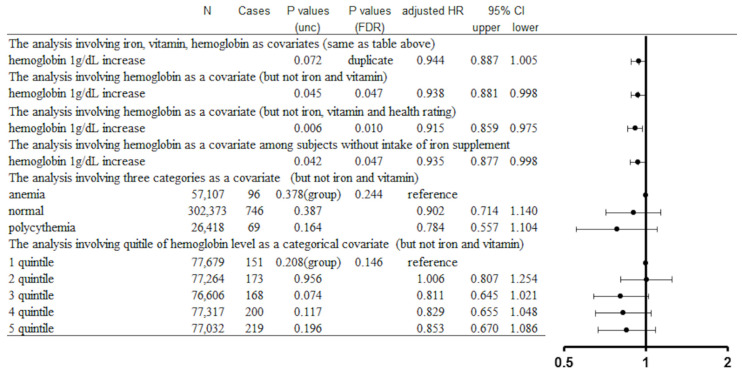
Statistical values for the associations between hemoglobin (defined as a continuous variable, according to disease status, or as quintiles) and all-cause parkinsonism incidence from the main analyses and sub-analyses.

We then assessed whether hemoglobin concentration is nonlinearly related to parkinsonism risk (specifically with an inverse U-shaped relationship). However, no significant relationship was found between hemoglobin concentration and parkinsonism risk whether subjects were divided into anemic, healthy, and polycythemic groups (*p* value of group differences = 0.378, FDR corrected *p* = 0.244) or into hemoglobin concentration quintiles (*p* value of group differences = 0.208, FDR corrected *p* = 0.146) (Figure 2).

### 3.4. Sensitivity Analyses

**Figure 3:**
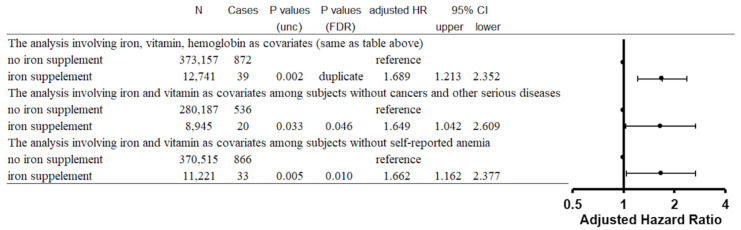
Statistical values for the associations between iron supplement intake and all-cause parkinsonism incidence from the main analyses and sensitivity analyses.

We then conducted sensitivity analyses to examine the effects of comorbidities on the associations between iron supplement intake with or without vitamin C or multivitamin intake and incident parkinsonism. First, we excluded subjects with cancers or any other serious diseases, and found that iron supplement intake was still associated with a significantly higher risk of parkinsonism (*p* = 0.033, FDR corrected *p* = 0.046, adjusted HR = 1.649, CI:1.042–2.609). Similarly, in analysis excluding subjects with self-reported anemia, iron supplement intake was associated with a significantly higher risk of parkinsonism (*p* = 0.005, FDR corrected *p* = 0.010, adjusted HR = 1.662, CI:1.162–2.377) (Figure 3).

### 3.5. Polygenic Risk Score Analysis

Finally, we conducted partial correlation analyses and multiple logistic regression analyses adjusting for covariates to investigate the effects of genetic predisposition to PD on iron supplement intake, vitamin C or multivitamin intake (multiple logistic regression analyses), and hemoglobin concentration (a partial correlation analysis). The results revealed that PRS of PD was significantly and negatively associated with hemoglobin concentration (*p* = 0.003, partial correlation coefficient = −0.006, N = 258,574) but not with iron supplement intake (*p* = 0.312, N = 265,848) and not with vitamin C or multivitamin intake (*p* = 0.114, N = 265,494).

## 4. Discussion

This study examined the effects of iron supplement intake with or without concomitant vitamin C or multivitamin intake and of hemoglobin concentration on parkinsonism risk using the large UK Biobank database. Specifically, we tested the 5 related hypotheses described in the Introduction. Hypothesis 1 was generally supported, as iron supplement intake was significantly associated with increased risk of incident parkinsonism. As described in the Introduction, previous findings have been inconsistent, with some studies reporting that high levels of dietary iron reduce PD risk [14,15] but another reporting faster disease progression [16]. To circumvent the possible causes of these discrepancies, the current study utilized a large-scale prospective cohort design and included only subjects without diagnosis of parkinsonism at baseline. Multivariable regress analyses revealed a robust association between iron supplement intake alone and increased risk of parkinsonism. In addition, hypothesis 5 was supported as this association remained after controlling for overall health rating and hemoglobin concentration as well after excluding subjects with cancer and other serious medical conditions. We also found that genetic predisposition to PD was not associated with iron intake. Collectively, these results suggest that the association of iron intake with parkinsonism is not explained by comorbid conditions requiring iron supplementation.

In contrast, hypothesis 3 was not supported as there was no significant or near-significant inverted U-shaped trend between hemoglobin concentration and parkinsonism risk, nor were there significant differences in risk between hemoglobin concentration groups stratified by clinical condition (polycythemic, anemia, and healthy) or quintiles. Previous studies have reported that low hemoglobin concentration and anemia are associated with elevated PD risk. The current study suggests these previous findings are not in conflict, as there were trends for greater parkinsonism risk among subjected reporting supplemental iron intake and those with low hemoglobin concentrations. We also found a negative association between iron intake and hemoglobin concentration, so the association between anemia and parkinsonism risk found in previous studies may have been influenced by iron supplement intake. However, even after excluding subjects reporting supplemental iron intake, hemoglobin level was still negatively correlated with parkinsonism risk (hypothesis 4 was confirmed). Furthermore, exclusion of overall health rating as a covariate, which was strongly associated with parkinsonism risk, strengthened the association between low hemoglobin concentration and parkinsonism risk. These alterations may suggest the association between hemoglobin levels and the risk of parkinsonism may be mediated by many factors.

Numerous studies have implicated iron dysregulation in PD pathogenesis. For instance, iron accumulation was detected in the post-mortem SN of PD patients [27] and a neuroimaging study found that iron accumulation was associated with the clinical severity of PD [28]. Infusion of excess iron into the mouse brain also induced oxidative stress, which is considered a major pathomechanism for neurodegeneration in PD [29]. Further, iron supplementation during the neonatal period resulted in motor dysfunction during adulthood, and this effect was associated with higher iron content, increased oxidative stress, and low dopamine content in the SN of model mice [30,31]. Treatment with exogenous iron chelators such as deferiprone and hinokitiol have also been reported to inhibit PD progression [32]. The biological function of iron relies on its low redox potential, which allows for the reversible transition from Fe^2+^ to Fe^3+^ to drive electron transfer reactions [33]. However, in the presence of oxygen and hydrogen peroxide, these redox reactions generate highly reactive hydroxyl radicals [33] that can cause DNA and protein damage, lipid peroxidation, and ultimately neuronal cell death [33], as well as induce mitochondrial dysfunction and catalyze α-synuclein misfolding, aggregation, and accumulation in the SN [34,35]. Iron is normally sequestered by metalloproteins, which allows electron transfer reactions to occur without reactive oxygen species production [33]. This sequestration may explain why free iron intake can enhance parkinsonism risk, whereas high iron in hemoglobin may not.

Hypothesis 2 that the risk of parkinsonism due to iron supplement intake can be mitigated by simultaneous vitamin C intake was neither confirmed nor disproven. This hypothesis was based on previous findings that participants consuming higher levels of iron and vitamin C from food are not a higher PD risk [17]. The group taking vitamin C or multivitamins and iron supplements at the same time tended to have a lower parkinsonism risk than the group taking iron supplements alone, and this latter group tended to have a higher PD risk than the group not taking iron supplements, but both differences were not significant. This was also the case when groups were defined by whether they took vitamin C or a multivitamin alone, rather than vitamin C or a multivitamin. Whether the association between high dietary iron (nonheme iron) and higher PD risk shown in previous studies can be moderated by concomitant vitamin C intake requires additional studies with larger or more specific cohorts.

One future research topic is whether there is an interaction effect between a genetic risk of hemochromatosis and iron supplementation intake. Hemochromatosis is a disorder in which extremely high levels of iron build up in the tissues. Perhaps, excessive iron intake is a risk factor, especially for those who have this genetic risk. The sample size in this study is large enough to detect the main effect of iron supplement intake but not large enough to investigate interesting research topics, such as whether iron supplementation presents a risk in people with minor genetic polymorphisms or whether an interaction exists between genetic polymorphisms and iron supplementation intake. These topics should be investigated in future studies.

This study has several limitations, including the prospective observational design. Although we performed sensitivity analyses and statistically corrected for numerous potential covariates, it is still not possible to completely exclude the possibility that subjects took iron supplements to address some other condition that influences parkinsonism risk. Similarly, hemoglobin levels may be higher or lower than normal due to factors that influence parkinsonism risk. Ultimately, the influence of iron intake needs to be confirmed in randomized controlled trials. The present study also did not consider differences in dietary iron as no such information was available in the UK Biobank from the sufficient sample size currently. It may be possible to assess the impact of dietary iron in future years by maintain dietary records and allowing time for the accumulation of more parkinsonism cases. Finally, the question regarding iron supplement intake did not distinguish between nonheme iron and heme iron. The relative risks conferred by these two forms of iron warrant additional study.

In conclusion, iron supplement intake was associated with greater risk of developing incident parkinsonism. This relationship is statistically robust, was obtained using a large sample number, and was still significant after correction for a variety of confounding factors and removal of patients with cancers and other medically serious conditions. No evidence was obtained that the relationship between hemoglobin and parkinsonism risk was nonlinear. The associations of iron supplement intake and low hemoglobin levels with parkinsonism risk appeared to occur simultaneously. The association between greater genetic risk of PD and lower hemoglobin concentration level may partly explain this phenomenon. The association with iron supplement intake is consistent with the observation that excessive iron intake accelerates PD progression [9]. Preliminary evidence is accumulating that iron chelators can slow PD progression [36]. Nevertheless, iron is an essential element for the organism, and iron deficiency has been reported as a risk factor for movement dysfunctions [37]. Iron supplements are therefore considered essential for iron deficiency, and adjustments to such supplements should be appropriate to the iron deficiency condition. Moreover, as noted in the Introduction section, the results of the observational studies are mixed. Future interventional studies are needed to examine whether iron supplement intake restrictions are protective toward PD.

## Figures and Tables

**Table 1:** Baseline covariate values for participants with and without subsequent incident parkinsonism.

	No Incident Parkinsonism (*n* = 384,987)	Incident Parkinsonism (*n* = 911)
	Mean (SD)	Mean (SD)
Age	56.11 (8.08)	62.9 (5.63)
Townsend deprivation index	−1.4 (3.02)	−1.36 (3.02)
Education level	14.35 (5.07)	13.82 (5.33)
BMI	27.33 (4.71)	27.49 (4.4)
MET *	31.87 (35.7)	29.72 (34.43)
Systolic BP	137.45 (18.52)	140.49 (19.04)
Alcohol unit	16.65 (19.83)	15.87 (19.69)
	Number (%)	Number (%)
Number of males	183,293 (47.6%)	595 (65.3%)
Household income		
(a) Less than £18,000	83,504 (21.7%)	334 (36.7%)
(b) £18,000–£30,999	97,357 (25.3%)	282 (31%)
(c) £31,000–£5,1999	101,774 (26.4%)	172 (18.9%)
(d) £52,000–£100,000	80,701 (21%)	93 (10.2%)
(e) More than £100,000	21,651 (5.6%)	30 (3.3%)
Currently employed	235,548 (61.2%)	283 (31.1%)
Number of people in the household		
(a) 1	73,174 (19%)	205 (22.5%)
(b) 2	175,574 (45.6%)	536 (58.8%)
(c) 3	60,534 (15.7%)	102 (11.2%)
(d) ≤4	75,705 (19.7%)	68 (7.5%)
Overall health (4 levels)		
(a) Poor	15,193 (3.9%)	74 (8.1%)
(b) Fair	76,588 (19.9%)	271 (29.7%)
(c) Good	225,432 (58.6%)	465 (51%)
(d) Excellent	67,774 (17.6%)	101 (11.1%)
Current smoking (3 levels)		
(a) No smoking	345,409 (89.7%)	854 (93.7%)
(b) Only occasionally	10,691 (2.8%)	13 (1.4%)
(c) On most or all days	28,887 (7.5%)	44 (4.8%)
Ethnicity (white)	367,918 (95.6%)	884 (97%)

* MET: metabolic equivalent of task hours. Physical activity level.

**Table 2:** Baseline variables of interest for participants with and without subsequent incident parkinsonism.

	No incident Parkinsonism (*n* = 384,987)	Incident Parkinsonism (*n* = 911)
	Mean (SD)	Mean (SD)
Hemoglobin concentration (g/dL)	14.20 (1.25)	14.34 (1.24)
	Number	Percent
Anemia, Polycythemia		
(a) Anemia	57,011 (14.8%)	96 (10.5%)
(b) Normal	301,627 (78.3%)	746 (81.9%)
(c) Polycythemia	26,349 (6.8%)	69 (7.6%)
Household income		
(a) Iron (X), Vitamin C + Multivitamin (X)	275,422 (71.5%)	664 (72.9%)
(b) Iron (O), Vitamin C + Multivitamin (X)	3203 (0.8%)	15 (1.6%)
(c) Iron (X), Vitamin C + Multivitamin (O)	96,863 (25.2%)	208 (22.8%)
(d) Iron (O), Vitamin C + Multivitamin (O)	9499 (2.5%)	24 (2.6%)

## Data Availability

Researchers can apply to use the UK Biobank resource (https://www.ukbiobank.ac.uk/) and access the data used.
